# Association of the cMIND diet with cognitive impairment in older adults: evidence from a 10-year nationwide study

**DOI:** 10.3389/fnut.2025.1716435

**Published:** 2026-01-13

**Authors:** Dahuan Cai, Yanxin Zeng, Xiaoping Xu, Mengliang Ye, Anchao Song, Min Chen

**Affiliations:** 1 School of Public Health, Chongqing Medical University, Chongqing, China; 2 The First Affiliated Hospital of Chongqing Medical University, Chongqing, China

**Keywords:** CLHLS, cMIND diet, cognitive impairment, longitudinal study, older adults

## Abstract

**Background:**

The protective effects of the Mediterranean-DASH Intervention for Neurodegenerative Delay (MIND) diet against cognitive impairment have been well-established in European and North American populations. However, due to differences in dietary patterns, evidence from cohort studies on the association between the currently adapted MIND diet and cognitive impairment in Chinese older adults remains limited, and a causal relationship has yet to be determined.

**Methods:**

A total of 8326 Chinese Longitudinal Healthy Longevity Survey (CLHLS) participants were included in this study. The Chinese version of the Mediterranean-DASH intervention for neurodegenerative delay (cMIND) diet scores ranged from 0 to 12 based on validated food frequency questionnaire responses. A Cox proportional hazards model was used to assess associations between cMIND diet and cognitive impairment in older adults. Restricted cubic spline plots were used to assess the linear relationship between cMIND and cognitive impairment.

**Results:**

The study found a significant nonlinear relationship between cMIND diet and cognitive impairment (P-overall < 0.001, P-non-linearity = 0.021). Compared with elderly people on a low-level cMIND diet, olders people on a high-level diet had a lower risk of developing cognitive impairment (HR = 0.79, *P* < 0.001). Subgroup analysis indicated that a high-level cMIND diet provided stronger protective effects for males (HR = 0.75, *P* < 0.001), rural residents (HR = 0.72, *P* < 0.001), and younger elderly individuals (HR = 0.71, *P* = 0.004).

**Conclusion:**

High adherence to the cMIND diet effectively reduces the risk of cognitive impairment in the Chinese elderly population, with enhanced protective effects observed specifically in males, those residing in rural areas, and younger elderly individuals.

## 1 Introduction

The world is currently facing the challenge of an aging population, and China, with the largest older population, is also one of the fastest-aging countries (1). According to data from the National Bureau of Statistics of China, by 2024, the population aged 65 and above in China has exceeded 220 million, representing 13.5% of the total population (2). This proportion is significantly higher than the global average (approximately 8%) and is projected to increase further in the future (3, 4). Against this background, the incidence of age-related diseases, particularly cognitive impairment, is expected to increase accordingly (5). Cognitive impairment is highly prevalent among older adults, encompassing a spectrum of conditions from mild cognitive impairment (MCI) to severe dementia (6). Currently, the overall prevalence of cognitive impairment among older adults is relatively high globally. Studies have shown that the prevalence of cognitive impairment among older adults in Europe has reached as high as 30.2% (7). In China, the prevalence of MCI among individuals aged 60 and above is 15.5%, while that of dementia is 6.0% and Alzheimer’s disease is 3.9% (8–10). Concurrently, cognitive impairment not only exerts a significant negative impact on patients and their families but also imposes a substantial burden on healthcare systems, thereby emerging as a serious public health issue (11). Based on this, it is necessary to identify the various risk factors influencing cognitive impairment in older adults and implement corresponding preventive measures to mitigate its harm.

Existing research has extensively explored the risk factors for cognitive impairment in older adults, including demographic factors such as age and gender, lifestyle-related factors such as smoking and physical activity, and physical and mental health factors such as body mass index (BMI) and activities of daily living (ADL) (5, 12). Among these, dietary status is considered to play a key role in the development of cognitive function in older adults (13). For example, research by Li et al. (14) and Jia et al. (15) has shown that dietary protein and vitamin B1 intake are positively correlated with cognitive function in older adults; and Hoscheidt et al. (16) and Madison et al. (17) indicated that the intake of different types of dietary fat may exert positive or negative effects on cognitive function in older adults. Based on these studies, researchers further explored the impact of different dietary patterns on cognitive impairment in older adults to address this issue (18–21). Among these, the Mediterranean-Dietary Approaches to Stop Hypertension (DASH) intervention for the neurodegenerative delay (MIND) diet, a combination of the Mediterranean diet and the DASH diet, has been proven to have a positive effect on improving cognitive function and preventing cognitive impairment in older adults (13, 22, 23). However, due to differences in dietary habits, the MIND diet is not applicable in China or even the entire East Asia region. Therefore, Huang and his team proposed the Chinese version of the Mediterranean-DASH intervention for the neurodegenerative delay (cMIND) diet (24). The cMIND diet, based on the MIND diet and modified to suit the dietary characteristics of Chinese people, aims to reduce the impact of cognitive impairment in older Chinese adults (25). Huang et al.’s study showed that high compliance with the cMIND diet is associated with a lower risk of cognitive impairment in older Chinese adults (24). However, this study is limited by its cross-sectional design and cannot effectively explore the causal relationship between the cMIND diet and cognitive impairment in Chinese older adults. Meanwhile, research in this field remains limited overall, particularly regarding the association between the cMIND diet and cognitive impairment in different subgroups of older adults.

Thus, this study aims to further explore the relationship between the cMIND diet and cognitive impairment in older Chinese adults, based on existing research findings and using data from the Chinese Longitudinal Health Longevity Survey (CLHLS). This will provide a theoretical basis for further research in related fields and the development of prevention and intervention strategies for cognitive impairment in this population.

## 2 Subjects and methods

### 2.1 Participants

The data for this study were sourced from the Chinese Longitudinal Health Longevity Survey (CLHLS). The CLHLS is a national prospective longitudinal study organized by the Peking University Centre for Healthy Aging and Development (CHADS) to investigate issues related to aging. The CLHLS has conducted seven follow-up surveys since the 1998 baseline survey. More information can be found in other related topics (26).

**Figure 1:**
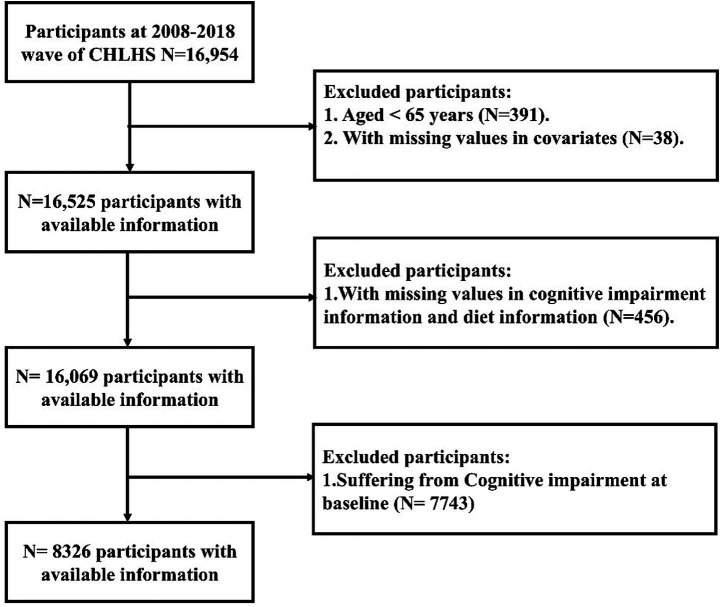
Participant screening flowchart.

This study utilized national follow-up data from four waves of the CLHLS from 2008 to 2018. First of all, this study excluded participants younger than 65 years of age (*n* = 391) and those lacking covariate information (*n* = 38). Furthermore, this study excluded participants lacking cMIND diet information (*n* = 456). Finally, this study excluded participants with cognitive impairment at baseline (*n* = 7,743). Ultimately, 8,326 participants were enrolled in this study (Figure 1).

### 2.2 Assessment of cMIND diet

The dietary data for this study were obtained from the simplified qualitative food frequency questionnaire (FFQ) of the CLHLS, covering 12 food groups: staple food type, staple food quantity, fresh vegetables, mushrooms, fresh fruits, cooking oil, fish, soybeans, nuts, garlic, tea, and sugar or sweets (27). The total score for the FFQ is 12 points. Among these, staple food and cooking oil are scored based on the types consumed, with scores of 1 or 0 points; the amount of staple food is scored based on weight, with a score of 1 or 0 points; the rest of the foods are scored based on frequency of consumption, with scores of 1, 0.5, or 0 points. The specific assessment criteria for FFQ are detailed in Supplementary Table S1. Concurrently, this study divided participants into three groups based on the tertiles of their cMIND diet scores, from low to high, namely T1, T2, and T3.

It is worth noting that the cMIND diet is not a simple translation of the MIND diet, but rather a recalibrated tool based on the dietary habits of the Chinese population, with multiple component revisions, deletions, and score threshold adjustments. Among them, Considering the low proportion of whole grains in the staple diet of the Chinese population, the cMIND diet replaces “whole grains” in the MIND diet with “types of staple food” and “amount of staple food”. Considering the low consumption of olive oil by the Chinese population, it is replaced by “edible oil” and divided into “vegetable oil” and “animal oil”. Considering that wine is rarely consumed, replace it with “tea”. Considering the low intake of butter/margarine, cheese and Fast fried foods by the Chinese population and the absence of similar substitutes, it is deleted. Delete red meat and poultry, as it is not specifically stated in the Dietary Guidelines for Chinese Residents. Otherwise, the other components of the cMIND diet are similar to the MIND diet, encouraging consumption of fresh vegetables, fruits, fish, soybeans, and nuts while discouraging sugar/sweets. The content and structural differences between the cMIND diet and the MIND diet are specifically shown in Supplementary Table S2. In addition, as China’s consumption of edible vegetable oils is dominated by seed-based oils such as soybean oil, rapeseed oil, peanut oil, and palm oil, while the consumption of fruit-based oils such as olive oil and coconut oil is extremely limited. Therefore, in this study, “vegetable oil” primarily refers to seed-based oil.

### 2.3 Assessment of cognitive impairment

This study assessed participants’ cognitive function using the Chinese Mini-Mental State Examination (cMMSE). In the CLHLS, the cMMSE comprises 24 items that cover five domains: orientation, registration, attention and calculation, recall, and language. The total score for the cMMSE is 30 points. Participants scoring less than 24 points are diagnosed with cognitive impairment. The specific assessment criteria for cMMSE are detailed in Supplementary Table S3. Currently, the cMMSE has demonstrated effectiveness in numerous prior studies, with a Cronbach’s alpha of 0.906 (11, 28).

### 2.4 Covariates

To obtain more accurate findings, the following covariates were included in this study based on the existing literature: gender (male, female), age (65–74, 75–89, ≥90 years), residence (urban, town, and rural), marital status (married and living with a spouse, separated/divorced/widowed/never married), currently smoking (yes, no), currently drinking (yes, no), physical exercise (yes, no), BMI (obese, overweight, normal, underweight), Activities of daily living (ADL) include basic activities of daily living (BADL) (yes, no) and instrumental activities of daily living (IADL) (yes, no). The specific assessment criteria for BADL and IADL are detailed in Supplementary Table S4.

### 2.5 Statistical analysis

Statistical analyses of the study were performed using Stata version 18.0 and R version 4.5.0. GraphPad Prism version 9.0 was used to visualize the results of the subgroup analyses. Descriptive statistics were used to summarize the baseline characteristics of the study population. Cox proportional hazard models were utilized to estimate hazard ratios (HRs) and their corresponding 95% confidence intervals (CI) for the association between the cMIND diet and cognitive impairment in older adults.

Restricted cubic splines were plotted to explore the potential nonlinear relationships between cMIND diet and cognitive impairment. The Bayesian Information Criterion (BIC) was applied to determine the optimal number of knots for the splines (29, 30). Subgroup analyses based on age and gender supported the robustness of the conclusions. Statistical significance was set at *p* < 0.05 for two-sided tests.

## 3 Results

### 3.1 Baseline characteristics

A total of 8,326 respondents were included in this study, of which 4,473 (53.7%) were males and 3,853 (46.3%) were females. The majority of older people live in rural areas (57.7%). The age range is between 65 and 113 years, with a median age of 81 years. By the end of the follow-up period, 25.6% of the participants had developed cognitive impairment (Table 1). The 2018 CLHLS sample shows good internal consistency for the ADL, with a Cronbach’s alpha of 0.82.

**Figure 2:**
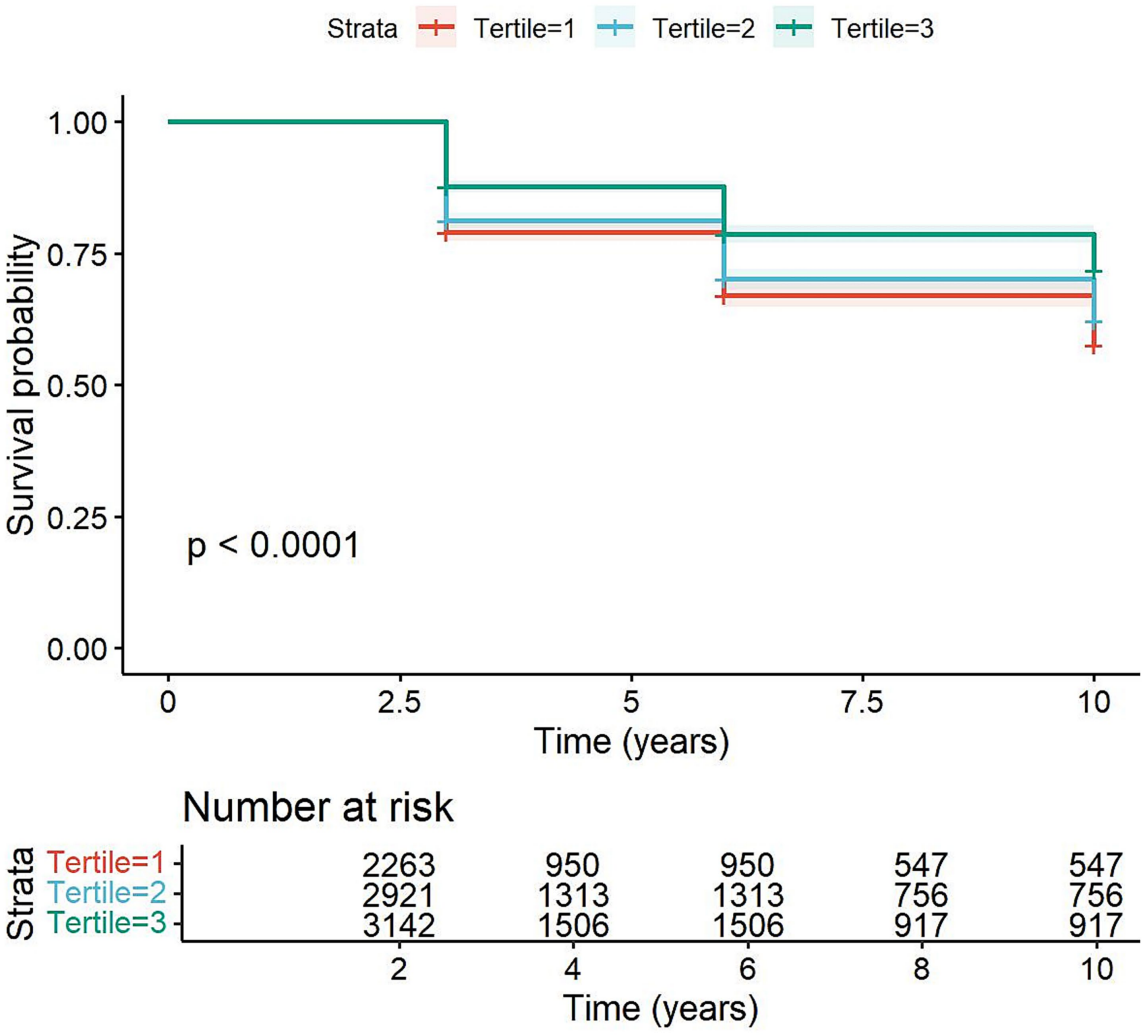
Kaplan–Meier survival curves for the three cMIND groups. Kaplan–Meier survival curves stratified according to cMIND score. The mean length of follow-up for participants was 6.65 years.

The results showed that the cMIND diet had significant differences in cognitive impairment, gender, age, education, residence, marital status, drinking, physical exercise, BMI, and instrumental activities of daily living (*p* < 0.001). From cMIND Tertile 1 to Tertile 3, the proportion of participants without cognitive impairment increased, while that with cognitive impairment decreased. This suggests that cMIND scores may be associated with cognitive impairment. The Kaplan–Meier survival curve is shown in Figure 2.

**Table 1:** Baseline characteristics of participants.

Characteristics	Total (*N* = 8,326)	Tertile 1	Tertile 2	Tertile 3	χ2_/*H*_	*p*-value
Cognitive impairment, *n*(%)						
Non-cognitive impairment	6,195 (74.4)	1,568 (69.3)	2,108 (72.2)	2,519 (80.2)	93.66	<0.001
Cognitive impairment	2,131 (25.6)	695 (30.7)	813 (27.8)	623 (19.8)		
Gender, *n*(%)						
Male	4,473 (53.7)	1,157 (51.1)	1,541 (52.8)	1775 (56.5)	16.93	<0.001
Female	3,853 (46.3)	1,106 (48.9)	1,380 (47.2)	1,367 (43.5)		
Residence, *n*(%)						
Urban	1851 (22.2)	224 (9.9)	519 (17.8)	1,108 (35.3)	639.96	<0.001
Town	1,676 (20.1)	373 (16.5)	712 (24.4)	591 (18.8)		
Rural	4,799 (57.6)	1,666 (73.6)	1,690 (57.9)	1,443 (45.9)		
Marital status, *n*(%)						
Married and living with a spouse	3,696 (44.4)	845 (37.3)	1,276 (43.7)	1,575 (50.1)	88.05	<0.001
Separated/divorced/widowed/never married	4,630 (55.6)	1,418 (62.7)	1,645 (56.3)	1,567 (49.9)		
Currently smoking, *n*(%)						
Yes	1852 (22.2)	488 (21.6)	638 (21.8)	726 (23.1)	2.23	0.328
No	6,474 (77.8)	1775 (78.4)	2,283 (78.2)	2,416 (76.9)		
Currently drinking, *n*(%)						
Yes	1714 (20.6)	426 (18.8)	568 (19.4)	720 (22.9)	17.05	<0.001
No	6,612 (79.4)	1837 (81.2)	2,353 (80.6)	2,422 (77.1)		
Physical exercise, *n*(%)						
Yes	3,162 (38.0)	628 (27.8)	1,048 (35.9)	1,486 (47.3)	221.75	<0.001
No	5,164 (62.0)	1,635 (72.2)	1873 (64.1)	1,656 (52.7)		
Body mass index, *n*(%)						
Underweight	5,215 (62.6)	1,332 (58.9)	1837 (62.9)	2046 (65.1)	192.56	<0.001
Normal	2,145 (25.8)	766 (33.8)	766 (26.2)	613 (19.5)		
Overweight	860 (10.3)	150 (6.6)	288 (9.9)	422 (13.4)		
Obese	106 (1.3)	15 (0.7)	30 (1.0)	61 (1.9)		
BADL, *n*(%)						
Yes	561 (6.7)	149 (6.6)	181 (6.2)	231 (7.4)	3.33	0.189
No	7,765 (93.3)	2,114 (93.4)	2,740 (93.8)	2,911 (92.6)		
IADL, *n*(%)						
Yes	4,057 (48.7)	1,232 (54.4)	1,431 (49.0)	1,394 (44.4)	53.57	<0.001
No	4,269 (51.3)	1,031 (45.6)	1,490 (51.0)	1748 (55.6)		
Age, median (25, 75%)	81 (72,90)	83 (74,91)	81 (73,90)	79 (71,89)	89.56	<0.001
Years of schooling, median (25, 75%)	1 (0,5)	0 (0,4)	0 (0,5)	3 (0,6)	300.35	<0.001

BADL, Basic Activities of Daily Living; IADL, Instrumental Activities of Daily Living.

According to the Kolmogorov–Smirnov normality test, Age and Years of schooling were found to be non-normally distributed. The Kruskal-Wallis H Test was used to analyze intergroup differences.

### 3.2 Association of cMIND diet with cognitive impairment in older adults

**Figure 3:**
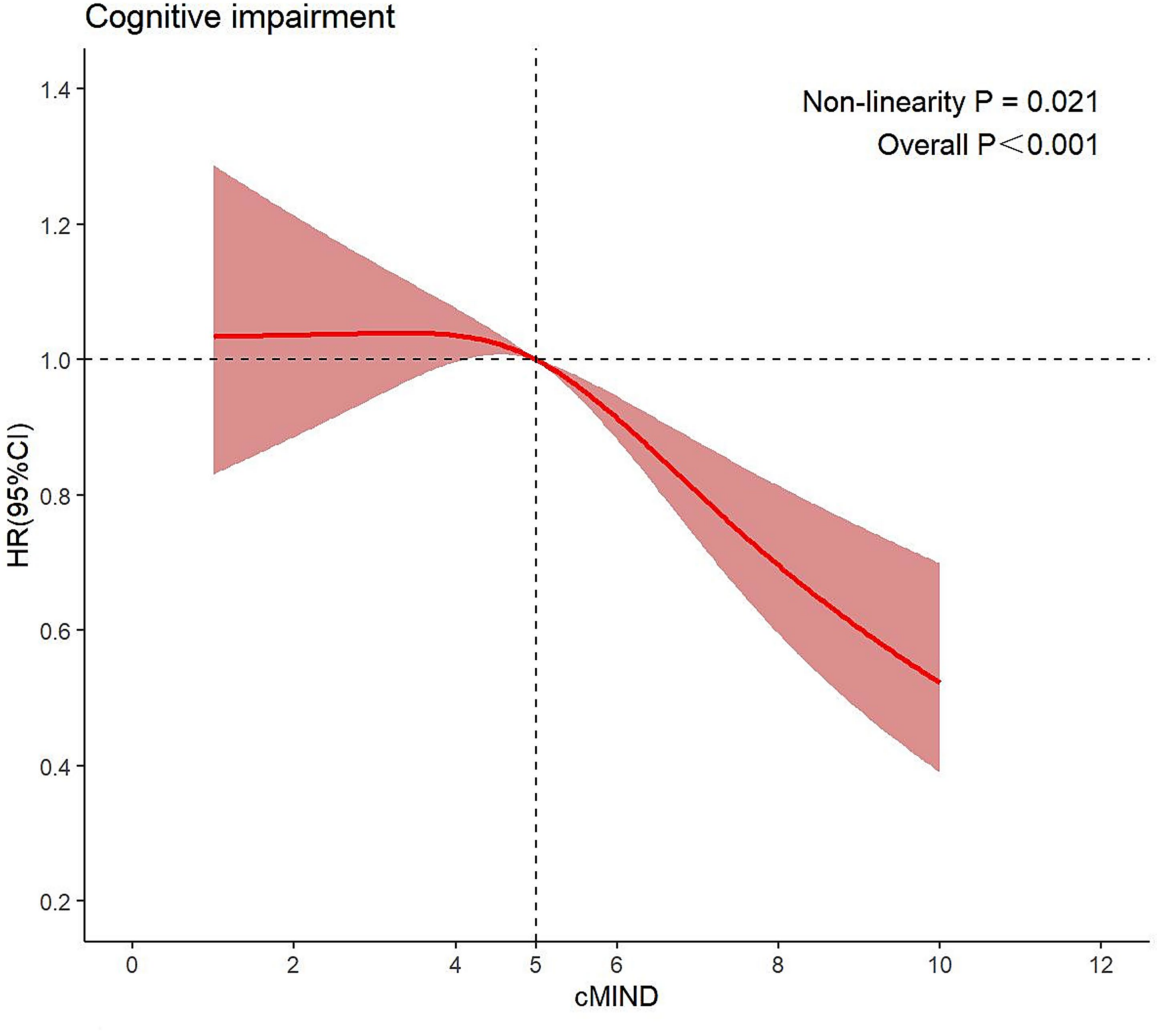
Restricted cubic spline curves for cMIND. HR refers to hazard ratio; CI refers to confidence interval. Shaded areas are 95% confidence intervals. The red line indicates the association between cMIND and cognitive impairment. The blue line indicates the association between years of schooling and cognitive impairment.

To further investigate the association between cMIND scores and years of schooling with cognitive impairment, we performed RCS analysis (Figure 3; Supplementary Figure S1). RCS shows that cMIND is nonlinearly associated with cognitive impairment in older adults (*P*-overall<0.001, *P-*non-linearity = 0.021), with an inflection point at 5. As shown in the figure, the relationship between the cMIND diet score and cognitive impairment risk was J-shaped. This pattern indicates that the association was not materially protective at lower adherence levels below a score of 5. In contrast, for individuals with scores above 5, each unit increase in the cMIND score was linked to a progressively greater reduction in risk. This finding suggests that dietary recommendations aiming to preserve cognitive function should prioritize helping individuals achieve at least a moderate level of adherence to the cMIND diet. Similarly, there is a nonlinear relationship between years of schooling and cognitive impairment (*P*-overall<0.001, *P-*non-linearity < 0.001), with a turning point at 1.

In Model 1 (as shown in Table 2), participants in the second and third cMIND tertiles had a lower risk of cognitive impairment compared to participants in the first tertile (HR = 0.89, *p* = 0.019; HR = 0.61, *p* < 0.001). Based on Model 1, Model 2 is adjusted for age, gender, schooling years, residence, currently smoking, currently drinking, physical exercise, and marital status. Compared with the first tertile of participants, the risk of cognitive impairment in the second tertile of participants was no longer statistically significant (HR = 0.97, *p* = 0.594), while the risk of cognitive impairment in the third tertile of participants remained lower (HR = 0.78, *p* < 0.001). In Model 3, which adjusted for all covariates, the risk of cognitive impairment remained lower in the third tertile of elderly people compared to the first tertile (HR = 0.79, *p* < 0.001).

**Table 2:** Association of baseline cMIND with the incidence of cognitive impairment risk.

Variables	Categories	Model 1		Model 2		Model 3	
		HR (95%CI)	*P*-value	HR (95%CI)	*P*-value	HR (95%CI)	*P*-value
cMIND group	Tertile 1	Reference		Reference		Reference	
	Tertile 2	0.89 (0.80–0.98)	0.019	0.97 (0.88–1.08)	0.594	0.98 (0.88–1.09)	0.684
	Tertile 3	0.61 (0.55–0.68)	<0.001	0.78 (0.70–0.87)	<0.001	0.79 (0.71–0.88)	<0.001
Gender	Male			Reference		Reference	
	Female			1.36 (1.22–1.51)	<0.001	1.34 (1.21–1.49)	<0.001
Age				1.03 (1.02–1.03)	<0.001	1.02 (1.01–1.03)	<0.001
Years of schooling				0.92 (0.91–0.94)	<0.001	0.92 (0.91–0.94)	<0.001
Residence	City			Reference		Reference	
	Town			1.17 (1.01–1.36)	0.035	1.19 (1.03–1.38)	0.019
	Rural			1.26 (1.10–1.43)	0.001	1.26 (1.11–1.44)	<0.001
Currently smoking	Yes			Reference		Reference	
	No			1.13 (0.99–1.28)	0.064	1.12 (0.99–1.27)	0.082
Currently drinking	Yes			Reference		Reference	
	No			0.99 (0.88–1.12)	0.925	0.99 (0.87–1.11)	0.819
Physical exercise	Yes			Reference		Reference	
	No			1.01 (0.92–1.11)	0.886	0.99 (0.90–1.09)	0.788
Marital status	Married			Reference		Reference	
	Separated/divorced/widowed/never married			1.07 (0.97–1.19)	0.172	1.06 (0.95–1.17)	0.301
Body mass index	Normal					Reference	
	Underweight					1.04 (0.95–1.15)	0.411
	Overweight					0.84 (0.72–0.99)	0.036
	Obese					1.11 (0.76–1.61)	0.596
BADL	Yes					Reference	
	No					0.89 (0.74–1.08)	0.241
IADL	Yes					Reference	
	No					1.26 (1.14–1.39)	<0.001

Model 1 was a crude model. Model 2 is adjusted for age, gender, years of schooling, residence, currently smoking, currently drinking, physical exercise, and marital status. Model 3 further adjusted for BMI, BADL, and IADL based on Model 2. BMI, Body Mass Index; BADL, Basic Activities of Daily Living; IADL, Instrumental Activities of Daily Living.

### 3.3 Differences in the association between cMIND diet and cognitive impairment in gender, age, and residence

**Figure 4:**
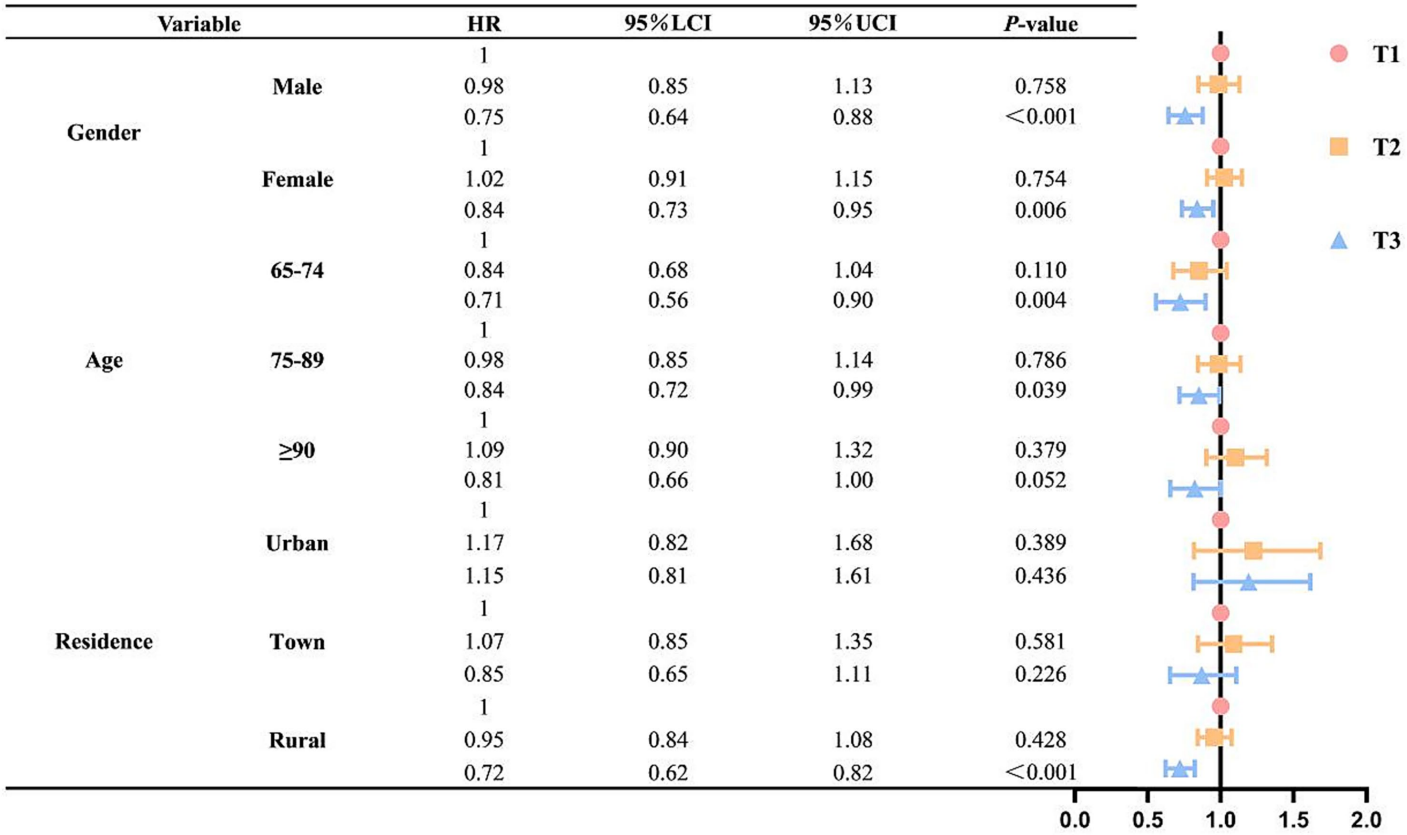
Association of gender, age, and residence subgroups of cMIND with the incidence of risk of cognitive impairment. Participants were then categorized into three groups (T1, T2, and T3) based on their scores. T1 refers to the lowest scoring of the three groups; T2 refers to the second highest scoring of the three groups; T3 refers to the highest scoring of the three groups.

To further examine the association between cMIND diet and cognitive impairment in different gender, age, and residence populations. We conducted subgroup analyses. The results of the study showed that in the cMIND high-level diet group, the cMIND diet was more protective for males compared to female older adults (HR = 0.75, *p* < 0.001). In the age subgroup, the cMIND diet was more protective in the 65–74 interval (HR = 0.71, *p* = 0.004). Whereas this association was not statistically significant in elderly aged 90 years and above (HR = 0.81, *p* = 0.052). In the residence subgroup, the cMIND diet had the strongest protective effect on the elderly in rural areas (HR = 0.72, *p* < 0.001). It was no longer significantly associated for the elderly in urban and town areas (HR = 1.15, *p* = 0.436; HR = 0.85, *p* = 0.226) (see Figure 4).

## 4 Discussion

This study assessed the association between the cMIND diet and cognitive impairment in older adults. The research results show that the cMIND diet is significantly negatively associated with cognitive impairment in older adults, with no linear relationship observed between them. Meanwhile, the risk of cognitive impairment in older adults is significantly reduced only in the high-level cMIND diet group. Moreover, this study found that the association between the cMIND diet and cognitive impairment in older adults differs across subgroups stratified by gender, age, and residence.

Prior to interpreting the aforementioned research findings, it is imperative to acknowledge the principal limitations of this study. Extant studies have demonstrated that accurately diagnosing cognitive impairment is a complex and rigorous process that often necessitates the integration of specialised neuropsychological assessment tools, clinical neurological examination, and biomarker testing as necessary. However, the purpose of using the MMSE in this study was not to diagnose cognitive impairment in participants but rather to provide initial screening and classification of participants with manifestations of cognitive impairment.

The results of this study indicate that the cMIND diet is associated with a reduced risk of cognitive impairment in older adults, with previous studies providing supporting evidence for our findings. Currently, the MIND diet has been proven to help reduce the risk of cognitive impairment in older adults (31, 32). For example, a prospective cohort study from the United States showed that high compliance with the MIND diet significantly reduced the risk of cognitive impairment in middle-aged and older adults. In this study, compared with the general adults, individuals with high compliance to the MIND diet exhibited a 15% lower risk of cognitive impairment (33). The cMIND diet is improved on the basis of the MIND diet, where it retains the benefits of the MIND diet while better aligning with the dietary habits of Chinese people (34). This effect primarily results from the cMIND diet’s enhancement of both overall health and dietary structure among older adults.

On the one hand, many existing studies have shown that the cMIND diet can have a wide-ranging impact on the physical and mental health of older adults. A cross-sectional study from China suggests that the cMIND diet can help reduce the likelihood of hypertension in older adults (25). Niu et al. (34) demonstrated that the cMIND diet had a significant protective effect on mental health in the older adults, significantly reducing the risk of affective disorders such as anxiety and depression. Moreover, the cMIND diet can also enhance the subjective well-being of the older adults to some extent (35). Meanwhile, existing studies have found that hypertension is often a significant risk factor for vascular cognitive impairment and dementia in the older adults (36). Anxiety and depression elevate the risk of cognitive impairment and dementia and may serve as a precursor to the two conditions (28). Additionally, subjective well-being has a significant protective effect on cognitive performance in older adults (37). Thus, we believe that the cMIND diet can improve the overall health of the older population somewhat by mitigating various physical and mental risk factors, thereby indirectly reducing the likelihood of cognitive impairment.

On the other hand, the cMIND diet also contributes to enhancing the dietary profile of the older population. Similar to the Mediterranean dietary pattern and the DASH diet, the cMIND diet is based on a plant-based diet and focuses particularly on the intake of healthy plant foods (e.g., whole grains, fresh vegetables, and fruits) and the exclusion of unhealthy plant foods (e.g., refined grains and sugar) (38). Previous studies have extensively explored the association between plant-based diet and cognitive function in older populations. The results show that a vegan diet alone is not associated with improved cognitive function in older adults, and that only healthy plant-based foods benefit cognitive function in older adults, while unhealthy plant-based foods are linked to an increased risk of cognitive impairment in older adults (39, 40). In addition, although the present study did not assess the type or amount of nutrients in the cMIND diet, findings from previous studies on nutrient-related mechanisms could provide theoretical support for the present study. For example, Ding et al. noted that fresh vegetables and fruits, key components of the cMIND diet and healthy plant foods, are abundant in diverse anti-inflammatory and antioxidant substances like vitamin C, vitamin E, and polyphenols, offering significant protection for cognitive functions (39). In contrast, the intake of unhealthy plant foods, such as sugar and refined grains, can result in memory and learning deficits, thus affecting cognitive function (41). Consequently, emphasizing adherence to the cMIND diet can significantly promote the intake of nutrients beneficial to cognitive function and cut the intake of detrimental nutrients, thereby enhancing cognitive function and aiding in preventing and treating cognitive impairment in older populations.

Based on the above discussion, this study also found that the association between the cMIND diet and cognitive impairment in older adults is not linear. Specifically, the risk of cognitive impairment decreases significantly only when older adults achieve high compliance with the cMIND diet, which is consistent with some previous studies (33, 42). We believe three main reasons underlie this phenomenon. To begin with, the cMIND diet is a comprehensive dietary pattern that emphasizes both the consumption of healthy foods and the restriction of unhealthy foods. Therefore, participants’ food intake is beneficial overall and protects cognitive function only when they achieve a certain level of compliance with the cMIND diet. Subsequently, certain beneficial components in the cMIND diet may have a dose–response relationship, meaning they only exert effects when intake levels and frequency reach a certain threshold. This requires high compliance with the cMIND diet. Thirdly, certain food combinations within the cMIND diet can elicit synergistic effects that amplify the protective benefits for cognitive function. For example, the synergistic effect of pickled vegetables consumed with garlic can reduce the risk of cognitive impairment in older populations (35). Finally, it should be noted that high compliance with the cMIND diet may also reflect the overall healthiness of participants’ lifestyle habits, which may have a more profound impact on their cognitive function.

Additionally, this study found that the association between the cMIND diet and cognitive impairment in older adults differs across subgroups stratified by gender, age, and place of residence. In the gender subgroup, this study’s results indicated a greater reduction in cognitive impairment risk among older men than among older women in the high-level group. We believe this is largely attributable to physiological differences between the sexes (43). Previous studies have shown that, despite controversy over gender differences in cognitive impairment, women are more prone to diseases such as dementia and Alzheimer’s than men and experience a greater decline in cognitive ability than men in a pathological context (10, 43, 44). This may be because estrogen regulates Amyloid-beta (Aβ) clearance and hippocampal neurogenesis, while its decrease during menopause may reduce neuroprotection (45–47). And this may indicate that women face a higher potential risk of cognitive impairment than men, resulting in lower health benefits from the cMIND diet than men. In the age subgroup, this study results revealed a greater reduction in cognitive impairment risk among older adults aged 65–74 years than among those aged 75–89 years in the high-level group. This may be because, on the one hand, age is a risk factor for cognitive impairment, and older elderly individuals typically face a higher risk (48). On the other hand, older elderly adults are more prone to various chronic diseases that affect cognitive ability, thereby masking the beneficial effects of dietary patterns on cognitive function, such as chronic kidney disease, chronic obstructive pulmonary disease (COPD), and multimorbidity (49–51). Furthermore, as older adults age, their physiological functions decline, and even with sufficient nutrient intake, they may not adequately absorb and effectively utilize these nutrients to improve cognitive function (52). In the residence subgroup, this study pointed out that, compared with older adults living in urban and towns, the risk of cognitive impairment was significantly reduced only among those living in rural areas within the high-level group. This may be attributed to differences in living environments. For example, Yu et al. reported that air pollution and noise may adversely affect cognitive abilities, with their synergistic effects being particularly pronounced in vulnerable groups, such as older adults (53). This may render urban and town older adults more susceptible to cognitive impairment and mask the beneficial effects of dietary patterns on cognitive function. Moreover, several social factors, such as food sources, processing methods, and accessibility, may also contribute to this phenomenon. In cities and towns, food is primarily sourced from external locations and undergoes extended periods of transportation, storage, and processing, along with more frequent use of food additives. However, in rural areas, food is more dependent on self-sufficiency and local cultivation, with relatively less reliance on food additives. Previous studies have shown that prolonged or poor storage conditions, such as light, heat, and oxidation, can lead to nutrient loss in foods (54). Meanwhile, many nutrients enriched by the cMIND diet, which positively affect cognitive performance in the older population, can be destroyed or lost at an accelerated rate due to food additives and processing. For example, vitamin B1 undergoes a nucleophilic reaction with sulfites, resulting in the loss of its activity (55); and vitamin C is extremely sensitive to high temperature, light, and oxygen, and is easily degraded by oxidation during food processing (56). Therefore, older adults in urban and town areas may struggle to achieve comparable levels of cognitive performance due to inadequate nutrient intake, even if they consume the same type and quality of food as their rural counterparts. However, it is important to note that the interpretation of subgroup differences in this study is exploratory and should be interpreted with caution, and the mediating role of these factors requires further investigation. In summary, older patients with cognitive impairment should be encouraged to adhere to the cMIND diet during prevention and clinical treatment, particularly those who are male, younger, and reside in rural areas.

In conclusion, based on the results of this study, from a policy perspective, promoting the cMIND diet has potentially positive implications for reducing the risk of cognitive impairment in older adults and warrants policymakers’ attention to enhance its popularity. However, the use of the cMIND diet in clinical and community practice needs to be explored more carefully and thoroughly. On the one hand, clinicians can therefore encourage older patients with cognitive impairment to follow it, but with due consideration of the impact of real-world factors such as personal tastes, economic conditions, cultural background and food availability on long-term adherence (57). On the other hand, community-based cognitive health programs promoting the cMIND diet need to be enhanced with nutritional education and social support to develop personalized programs in order to improve their adaptability and effectiveness in real-life scenarios (58). In addition, it is particularly important to note that significant health benefits on cognitive performance in the older population only occur when the cMIND diet score is 5 or higher, as suggested by the results of the restricted cubic spline. This indicates that the older population needs to maintain at least a moderate level of adherence to the cMIND diet. Thus, it is important for clinicians to use a cMIND diet score of >5 as a basic intervention goal and to use a score of 5 as a tool for assessing the risk of cognitive impairment; and for policymakers, high adherence to the cMIND diet should be emphasized during community interventions, and resources should be allocated toward moderately adherent populations to maximize intervention benefits.

In the end, there remains a need for improvement in the cMIND diet. Firstly, considering that the meat intake of the Chinese population in the traditional dietary pattern is significantly lower than that of the Western population, the cMIND diet does not include the assessment of red meat and does not distinguish between red meat and white meat. However, with the increasing consumption of meat in China, the importance of meat to the cognitive ability of the Chinese population continues to grow, and it is increasingly important to explore the role of different types of meat (59, 60). Thus, the lack of a comprehensive assessment of meat consumption patterns is indeed an important limitation of the cMIND diet that needs to be addressed in future studies. Secondly, the current cMIND diet also fails to make a valid distinction between the health effects of seed-based oils and fruit-based oils. Currently, extensive research indicates that fruit-based oils, such as coconut oil and olive oil, can help improve cognitive function in older populations (61–63). Simultaneously, few studies have demonstrated the positive effects of seed-based oils on cognitive function in the older population. Therefore, the practice of generally attributing health effects to vegetable oils also has limitations. Based on this, future studies need to more carefully classify vegetable oils in the cMIND diet to more clearly reflect the health effects of different vegetable oils on cognitive function in the older population.

## 5 Innovations and limitations

This study has the following advantages. First of all, the data used in this study are nationally representative. This prospective study helps to establish a temporal association between the cMIND diet and cognitive impairment, providing evidence for the causal direction of the association. Finally, this study provides further evidence for the association between the cMIND diet and cognitive impairment in Chinese older adults and examines the impact of the cMIND diet in different Chinese older adult populations through subgroup analysis.

Nevertheless, this study also has certain limitations. To begin with, there is a high competing risk in the older population, which may lead to underestimation of the association with cognitive impairment. Although this issue has been mitigated by sensitivity analyses in this study, survival bias may still exist. Subsequently, the assessment of cognitive functional status in this study relied primarily on self-report questionnaires and lacked clinical diagnostic and biomarker data. Therefore, it was not able to establish a potential biological mechanistic link between cMIND scores and cognitive health status. Furthermore, due to data limitations, some covariates were not included, potentially leading to confounding bias. Moreover, the cMIND diet scores were calculated from baseline dietary data only and did not take into account subsequent dietary changes in the participants, which may introduce bias. Finally, the existing cMIND diet evaluation system requires further optimization. On the one hand, the study only considered the frequency of food, and food portion size was not taken into account. On the other hand, complete meat consumption patterns were not included in this study, and red, white, and other meats were not scored differently. Therefore, the cMIND diet needs refinement in these areas to ensure increased rigor in future studies.

## 6 Conclusion

This study suggests that adherence to the cMIND diet may effectively reduce the risk of cognitive impairment in older adults. It is recommended that policymakers enhance the promotion of the cMIND diet and that physicians encourage older patients with cognitive impairment to adopt the cMIND diet, with particular emphasis on older men, younger older adults, and older adults in rural areas.

## Data Availability

Publicly available datasets were analyzed in this study. This data can be found here: https://opendata.pku.edu.cn/dataverse/CHADS.
