# Correction: Association of the cMIND diet with cognitive impairment in older adults: evidence from a 10-year nationwide study

**DOI:** 10.3389/fnut.2026.1793751

**Published:** 2026-02-02

**Authors:** Dahuan Cai, Yanxin Zeng, Xiaoping Xu, Mengliang Ye, Anchao Song, Min Chen

**Affiliations:** 1 School of Public Health, Chongqing Medical University, Chongqing, China; 2 The First Affiliated Hospital of Chongqing Medical University, Chongqing, China

**Keywords:** CLHLS, cMIND diet, cognitive impairment, longitudinal study, older adults

Author Xiaoping Xu was erroneously omitted as equal contributing author. Xiaoping Xu should be listed as a first co-author (with †).

The original version of this article has been updated.

