# Supplementary material for: Association of the cMIND diet with cognitive impairment in older adults: evidence from a 10-year nationwide study
Source: Front Nutr. 2026 Jan 13;12:1716435. doi: 10.3389/fnut.2025.1716435 (PMC12834752; doi:10.3389/fnut.2025.1716435)
Supplement: Supplementary file 1 [file Table_1.DOCX]

Contents

1. Table S1 The simplified Food Frequency questionnaire and Scoring of cMIND diet 01
2. Table S2 Comparison of the content and structure of the cMIND diet and the MIND diet 02
3. Table S3 The mini-mental state examination (MMSE) of Chinese version in the CLHLS 04
4. Table S4 Assessment of Activities of Daily Living (ADL) in the CLHLS 05

5. Figure S1 Restricted cubic spline curves for years of education 06

Table S1 The Simplified Food Frequency questionnaire and Scoring of cMIND diet

| Components | Score | | |
| --- | --- | --- | --- |
|  | 0 | 0.5 | 1 |
| Type of staple food | Rice/wheat |  | Whole grains |
| Amount of staple food | <250g or >400g/day |  | 250g-400g/day |
| Fresh vegetables | ≤2 servings/week | 3–5/week | ≥6 servings/week |
| Mushroom or algae | ≤1 meal/week | 1-3/week | ≥4 meals/week |
| Fresh fruit | ≤2 servings/week | 3-5/week | ≥6 servings/week |
| Cooking oil | Animal oil |  | Vegetable oil |
| Fish | <1/month | 1-3/month | ≥1 meal/week |
| Food made from beans | <1 meal/week | 1-3 meal/week | ≥4 meals/week |
| Nut products | <1 serving/week | 1-4/week | ≥5 servings/week |
| Garlic | <1 meal/week | 1-3/week | ≥4 meals/week |
| Tea | Not almost every day | Other types of tea(almost every day) | Green tea(almost every day) |
| Sugar | ≥2 servings/week | 1/month-1/week | <1 serving/month |
| Note: Total score ranging from 0 to 12. | | | |

Table S2 Comparison of the content and structure of the cMIND diet and the MIND diet

| Number | MIND diet | | Number | cMIND diet | |
| --- | --- | --- | --- | --- | --- |
|  | **Components** | **Options** |  | **Components** | **Options** |
| 1 | Whole grains | <1 serving/day | 1 | Type of staple food | Rice/wheat |
|  |  | 1-2 servings/day |  |  | Whole grains |
|  |  |  | 2 | Amount of staple food | <250gor >400g/day |
|  |  | ≥3 servings/day |  |  | 250g-400g/day |
| 2 | Green leafy vegetables | ≤2 servings/week | 3 | Fresh vegetables | ≤2 servings/week |
|  |  | 3 to 5 servings/week |  |  | 3–5/week |
|  |  | ≥6 servings/week |  |  | ≥6 servings/week |
| 3 | Other vegetables | <5 servings/week | 4 | Mushroom or algae | ≤1 meal/week |
|  |  |  |  |  | 1-3/week |
|  |  | 5 to 6 servings/week |  |  | ≥4 meals/week |
|  |  |  | 5 | Garlic | <1 meal/week |
|  |  | ≥1 servings/day |  |  | 1-3/week |
|  |  |  |  |  | ≥4 meals/week |
| 4 | Berries | <1 serving/week | 6 | Fresh fruit | ≤2 servings/week |
|  |  | 1 serving/week |  |  | 3-5/week |
|  |  | ≥2 servings/week |  |  | ≥6 servings/week |
| 5 | Olive oil | Not primary oil | 7 | Cooking oil | Animal oil |
|  |  | Primary oil used |  |  | Vegetable oil |
| 6 | Fish  (not fried) | Rarely | 8 | Fish | <1/month |
|  |  | 1–3 meals/month |  |  | 1-3/month |
|  |  | ≥1 meals/week |  |  | ≥1 meal/week |
| 7 | Beans | <1 meal/week | 9 | Food made from beans | <1 meal/week |
|  |  | 1–3 meals/week |  |  | 1-3 meal/week |
|  |  | >3 meals/week |  |  | ≥4 meals/week |
| 8 | Nuts | <1 serving/month | 10 | Nut products | <1 serving/week |
|  |  | 1 serving/month to <5 servings/week |  |  | 1-4/week |
|  |  | >5 servings/week |  |  | ≥5 servings/week |
| 9 | Wine | >1 glass/day or never | 11 | Tea | Not almost  every day |
|  |  | 1 glass /month-  6 glasses/week |  |  | Other types of tea(almost every day) |
|  |  | 1 glass/day |  |  | Green tea(almost every day) |
| 10 | Pastries  and sweets | ≥7 servings/week | 12 | Sugar | ≥2 servings/week |
|  |  | 5–6 servings/week |  |  | 1/month-1/week |
|  |  | <5 servings/week |  |  | <1 serving/month |
| 11 | Butter/  margarine | >2 servings/day |  | | |
|  |  | 1–2 servings/day |  |  |  |
|  |  | <1 serving/day |  |  |  |
| 12 | Cheese | ≥7 servings/week |  |  |  |
|  |  | 1–6 servings/week |  |  |  |
|  |  | <1 serving/week |  |  |  |
| 13 | Red meat and products | ≥7 meals/week |  |  |  |
|  |  | 4–6 meals/week |  |  |  |
|  |  | <4 meals/week |  |  |  |
| 14 | Poultry (not fried) | <1 meal/week |  |  |  |
|  |  | 1 meal/week |  |  |  |
|  |  | ≥2 meals/week |  |  |  |
| 15 | Fast fried foods | ≥4 times/week |  |  |  |
|  |  | 1–3 times/week |  |  |  |
|  |  | <1 time/week |  |  |  |
| Note: The MIND diet contains 15 components and the cMIND diet contains 12 components. | | | | | |

Table S3 The mini-mental state examination (MMSE) of Chinese version in the CLHLS

| Domins | Questions | Scores |
| --- | --- | --- |
| Orientation | 1. What time of day is it right now (morning, afternoon, evening)? | 1 |
|  | 2. What is the month (Western or Chinese calendar) right now? | 1 |
|  | 3. What is the date (Chinese calendar day and month) of the mid-autumn festival? | 1 |
|  | 4. What is the season right now, spring, summer, fall, winter? | 1 |
|  | 5. What is the name of this district or town? | 1 |
|  | 6. Please name as many kinds of food as possible in 1 minute. | 7 |
| Registration | 1. I am now going to test your memory. I will mention three objects. Please repeat these three objects (table, apple, clothes). | 3 |
| Attention and calculation | 1. I will ask you to spend 3 dollars from 20 dollars, then you must spend 3 dollars from the number you arrived at and continue to spend 3 dollars until you are asked to stop. | 5 |
|  | 2. Ask the interviewee to draw the figure on B Card | 1 |
| Recall | 1. Please repeat the three words (in any order) that I asked you to repeat a little while ago. | 3 |
| Language | 1. Give the interviewee a pen and then a watch and ask what these objects are called(pen, watch). | 2 |
|  | 2. I will now ask you to repeat the following sentence:‘What you plant, what you will get.’ | 1 |
|  | 3. I will give you a piece of paper. You must take the paper using your right hand, fold it in the middle using both hands, and place the paper on the floor. | 3 |
| Note: Total score ranging from 0 to 30. Scores from 24 to 30, normal cognitive function. Scores from 0 to 23, cognitive impairment。 | | |

Table S4 Assessment of Activities of Daily Living (ADL) in the CLHLS

| Domins | Questions | Frequency |
| --- | --- | --- |
| BADL | 1. Bathing–either sponge bath, tub bath, shower or washing the body. | 1. Yes, independently;  2. Yes, but need some assistance;  3. No, can’t. |
|  | 2. Dressing–gets clothes from closets and drawers–including underwear, outer garments and fasteners (including suspenders, if worn). |  |
|  | 3. Toilet–going to the toilet; cleaning oneself afterwards. |  |
|  | 4. Indoor Transfer |  |
|  | 5. Continence |  |
|  | 6. Eating |  |
| IADL | 1. Can you visit your neighbors by yourself? |  |
|  | 2. Can you go shopping by yourself? |  |
|  | 3. Can you cook a meal by yourself whenever necessary? |  |
|  | 4. Can you wash clothing by yourself whenever necessary? |  |
|  | 5. Can you walk continuously for 1 kilometer at a time by yourself? |  |
|  | 6. Can you lift a weight of 5kg, such as a heavy bag of groceries? |  |
|  | 7. Can you continuously crouch and stand up three times? |  |
|  | 8. Can you take public transportation by yourself? |  |
| Note: If participants are unable to perform one or more items related to BADL without assistance, they will be classified as BADL disability; Similarly, if participants are unable to perform one or more items related to IADL without assistance, they will be classified as IADL disability. | | |


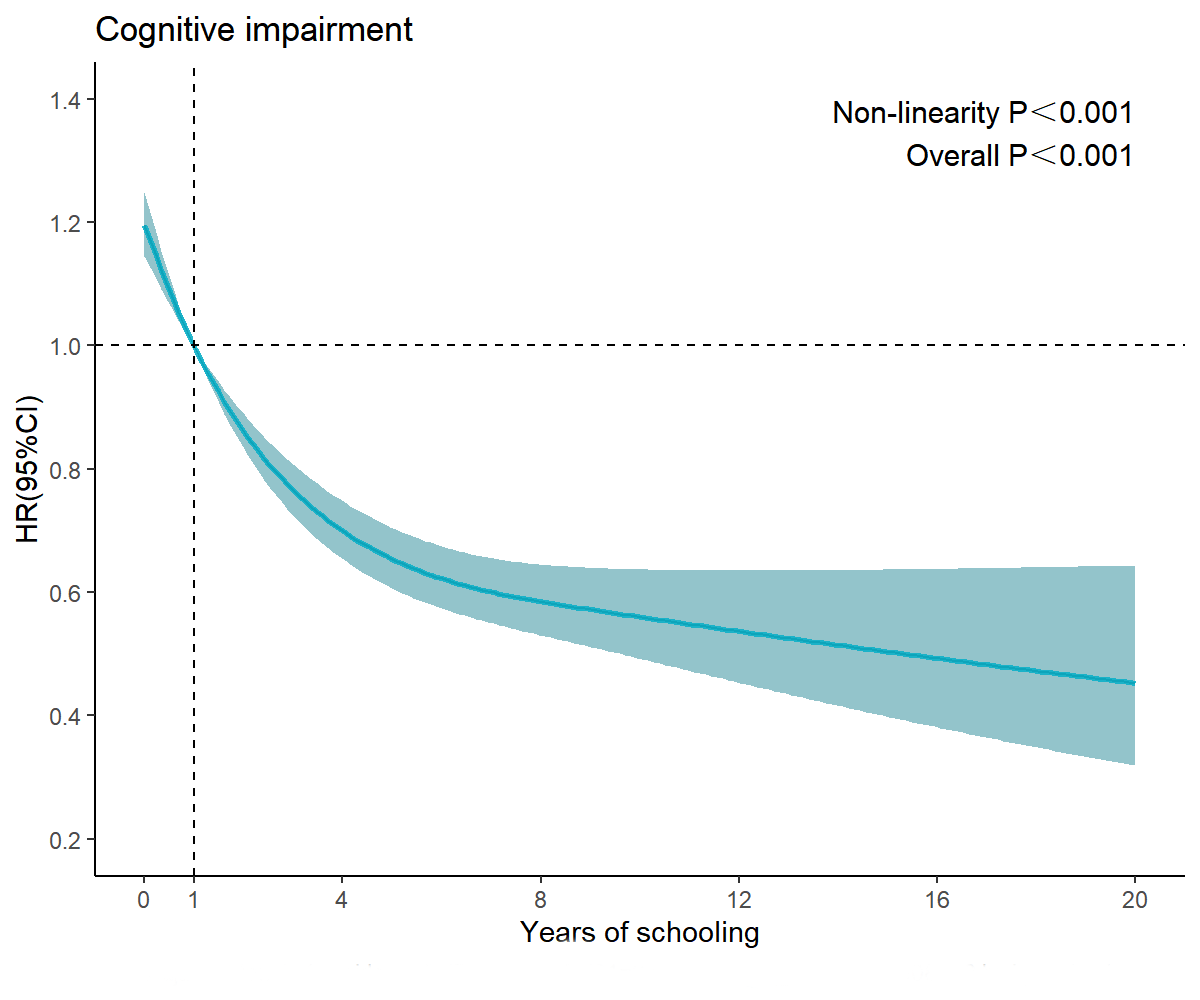


Figure S1 Restricted cubic spline curves for years of education.

Note: HR refers to hazard ratio; CI refers to confidence interval. Shaded areas are 95% confidence intervals. The blue line indicates the association between years of schooling and cognitive impairment.
